# Erythema nodosum leprosum as the initial presentation of lepromatous leprosy

**DOI:** 10.1016/j.jdcr.2024.07.034

**Published:** 2024-08-28

**Authors:** Grace Hingtgen, Rafael Mojica, Norman Beatty, Kiran Motaparthi

**Affiliations:** a University of Florida College of Medicine, Gainesville, Florida; b Department of Dermatology, University of Florida College of Medicine, Gainesville, Florida; c Division of Infectious Diseases & Global Medicine, University of Florida College of Medicine, Gainesville, Florida

**Keywords:** acid-fast bacilli, erythema nodosum leprosum, immune complex, leprosy, lymphohistiocytic infiltrate, neutrophils, type 2 reaction

## Introduction

Erythema nodosum leprosum (ENL), also known as type 2 immunologic reaction or Lepra reaction, is an immunologic complication of leprosy. ENL presents with painful subcutaneous nodules, vesicles, pustules, or ulcers. ENL is a systemic reaction that includes fever, neuritis, and involvement of other organs.^1^ This report describes ENL as the initial presentation of lepromatous leprosy (LL), without prior lepromatous symptoms, skin findings, or treatment. This presentation is uncommon, as ENL typically occurs after treatment for LL or borderline lepromatous leprosy (BL).^2^

## Case report

**Fig 1:**
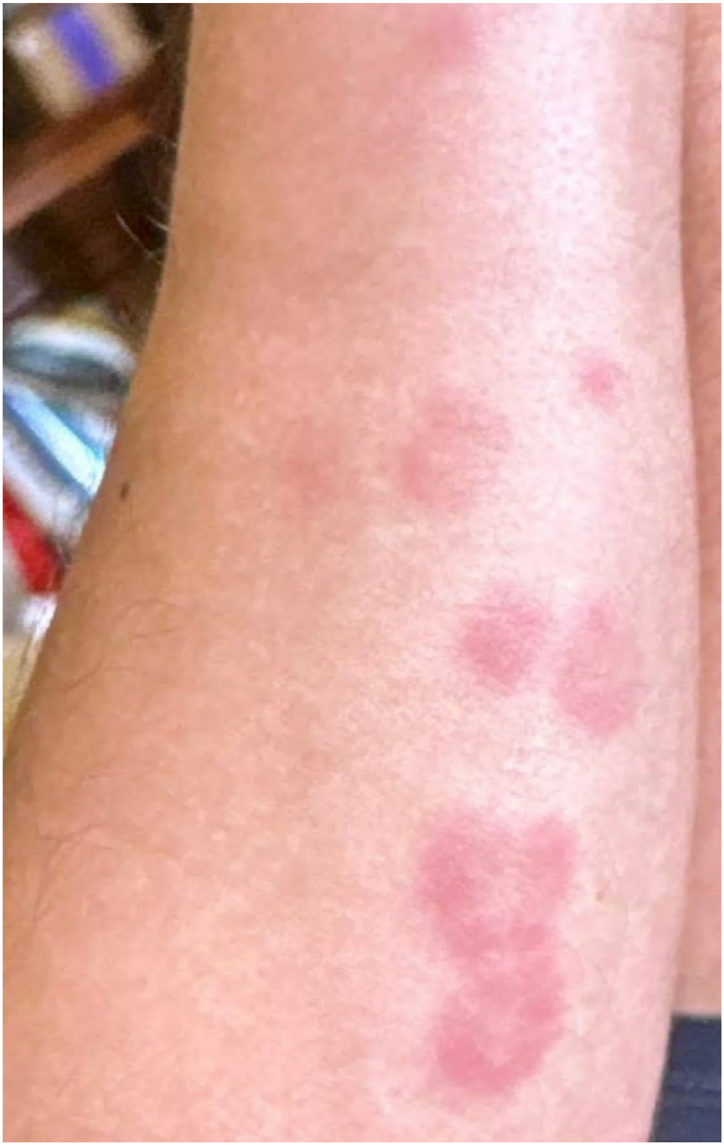
Erythematous, edematous papules and nodules with scattered erythematous patches on the upper extremities.

**Fig 2:**
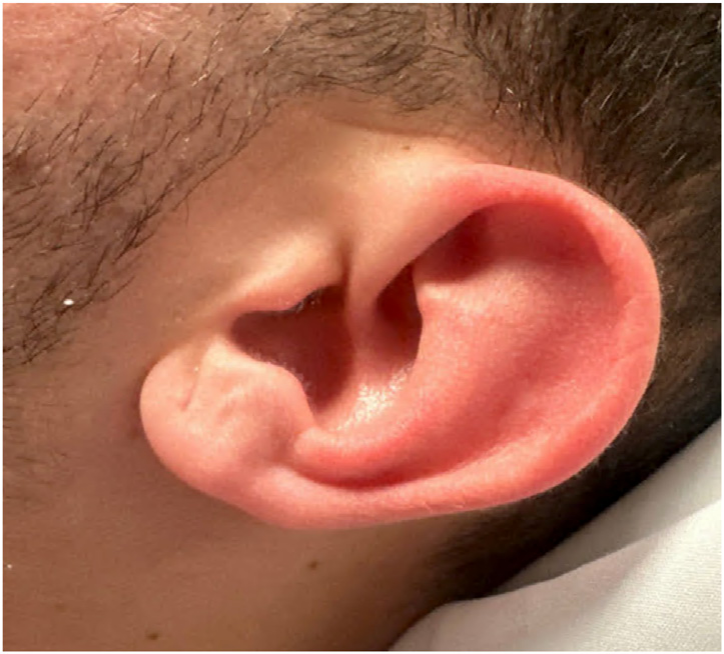
Nontender fullness of the auricle with lack of nodularity.

A 22-year-old man emigrated from Cuba via Nicaragua 2 years prior. Shortly after emigration, he experienced recurrent fevers, myalgias, night sweats, and inguinal lymphadenitis. Courses of doxycycline or systemic steroids prescribed by nondermatologists in an urgent care setting produced improvement followed by recurrence. Two months preceding presentation, he developed recurrent fevers peaking at 103.2 °F, inguinal lymphadenitis, paresthesias and weakness affecting the distal upper extremities, and a painful rash. Physical exam demonstrated erythematous, edematous papules and nodules without secondary change on the cheek and distal extremities (Figs 1 and 2). There were also scattered erythematous plaques and postinflammatory changes on the upper extremities. Additional examination revealed hyperlinearity and scaling of the left palm and several digits on the right hand and fullness of the ears without tenderness but no ulnar nerve thickening. Tender bilateral inguinal lymphadenitis was present. Neurologic exam demonstrated diminished grip strength in the left upper extremity, thenar and hypothenar atrophy, and decreased sensation to pinprick and light touch in the distal upper extremities. Fundoscopic exam was normal. Review of systems was notable for night sweats, photophobia, upper extremity weakness, arthralgia, and a single episode of testicular pain 1 month prior.

Laboratory values were significant for thrombocytopenia (100 × 10 E^3^/uL), euglycemia, and normal ferritin (194.9 ng/mL). Sex hormone-binding globulin (15 nmol/L) and total testosterone (299 ng/dL) were both low. Erythrocyte sedimentation rate (23 mm/hr) and C-reactive protein (258.38 mg/L) were elevated. Antinuclear antibody testing was negative. Complement and immunoglobulin levels were within normal limits. *Treponema pallidum* IgG and venereal disease research laboratory serologies were negative. Lyme, HIV, human T-lymphotropic virus I/II, and Epstein-Barr virus serologies were negative. HIV-1 RNA was not detected. Computed tomography imaging demonstrated enlarged bilateral inguinal lymph nodes but no lymphadenopathy elsewhere.

While initially broad, the differential diagnosis was narrowed to chronic conditions that could account for fever, lymphadenitis, nodules, and neuropathy. This limited differential diagnosis included ENL, borreliosis, and paraneoplastic findings due to lymphoma, specifically angioimmunoblastic T-cell lymphoma.

**Fig 3:**
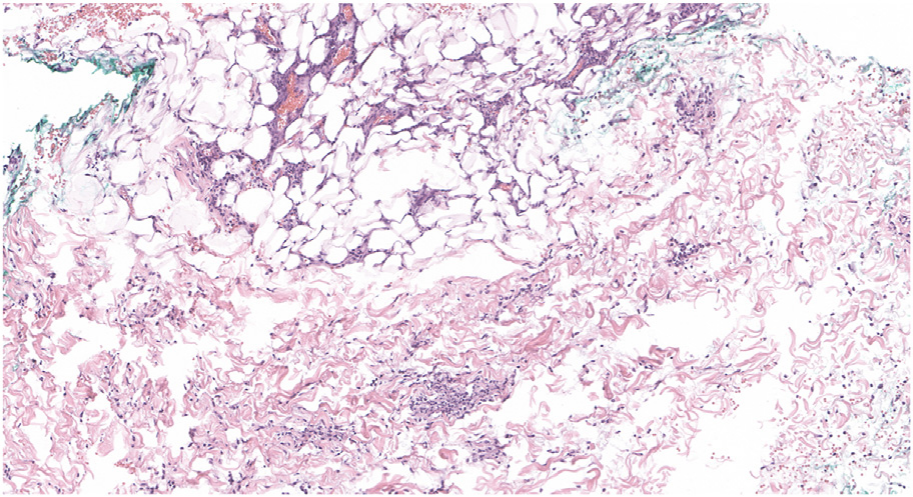
Skin punch biopsy demonstrating perivascular and interstitial lymphohistiocytic infiltrate with neutrophils. Endothelial swelling is seen, along with a subtle lobular panniculitis (H&E, 106× magnification).

**Fig 4:**
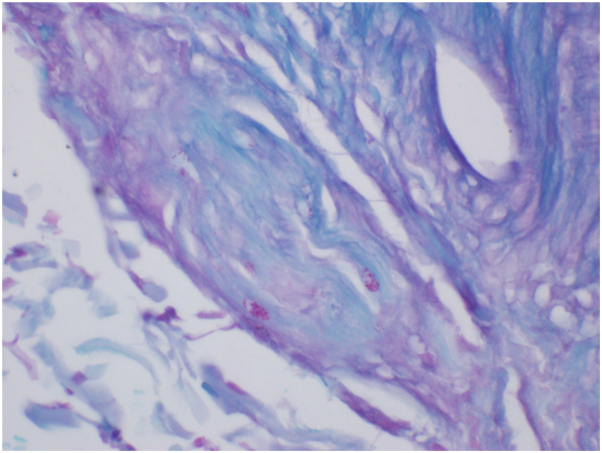
Numerous short acid-fast bacilli within a swollen small-caliber nerve (Fite-Faraco stain, 600× magnification).

Skin biopsies demonstrated a perivascular and interstitial lymphohistiocytic infiltrate with neutrophils. Additionally, there was prominent endothelial swelling of small and medium vessels, perineural and perieccrine inflammation, and a subtle lobular panniculitis (Fig 3). Fite-Faraco stain highlighted numerous short acid-fast bacilli in a perivascular, perineural, perieccrine, and interstitial distribution (Fig 4). Based on these findings, a diagnosis of ENL was made. Later, polymerase chain reaction of skin identified *M. leprae*. Treatment was initiated with pulse intravenous methylprednisolone 1 g IV daily for 3 days, with rapid resolution of systemic symptoms and cutaneous findings. Thereafter, treatment for the immunologic reaction included prednisone 20 mg daily tapered over 60 days, methotrexate 20 mg weekly, thalidomide 100 mg daily, and duloxetine 30 mg daily for neuropathy. Bactericidal treatment for LL included rifabutin, moxifloxacin, and minocycline at doses of 150 mg, 400 mg, and 100 mg monthly, respectively, with therapy planned for 2 years. Rifabutin was selected instead of rifampin to reduce the risk of drug interactions.

## Discussion

Given the average incubation period of 8 years in LL, this patient most likely became infected in Cuba. Despite achieving elimination of leprosy in 1993, according to the World Health Organization, new cases of leprosy are still described in Cuba. Clinicopathological presentations of leprosy range from LL to tuberculoid leprosy, with most patients classified as borderline tuberculoid or BL. Presentation is determined by the host’s immune response to *M. leprae* or *M. lepromatosis*.^3^ There are 2 main categories of immunologic reactions in leprosy: type 1 (reversal or upgrading reactions and downgrading reactions) and type 2 (ENL) reactions.^4^^,^^5^ Type 1 reactions can be observed in any form of leprosy and may occur before, during, or after therapy.^4^^,^^5^ Type 1 immunologic reactions are delayed cell-mediated type IV hypersensitivity reactions that present with increased erythema and edema of preexisting lesions and neuritis due to cellular immunity, but without systemic symptoms.^2^^,^^6^ In contrast, ENL is an immune complex-mediated type III hypersensitivity reaction observed in approximately 15.4 percent of patients with LL and 4.1 percent of patients with BL, usually following multidrug treatment.^7^, ^8^, ^9^ ENL presents with systemic inflammation in addition to *de novo* cutaneous lesions.^2^^,^^6^ The pathogenesis of ENL reflects neutrophilic invasion, immune complexes, pro-inflammatory cytokines, and a T cell-mediated response.^9^

The classic presentation of ENL includes painful, erythematous nodules, papules, and plaques often on the face and extremities.^6^ Untreated, recurrent episodes typically last 1-2 weeks but can persist chronically.^9^ Other morphologies include bullous, ulcerated, pustular, hemorrhagic, necrotic, and erythema multiforme-like (targetoid).^6^^,^^7^^,^^9^, ^10^, ^11^, ^12^ Systemic features include cervical or inguinal lymphadenitis, neuritis of distal nerves, arthritis, synovitis, iritis or uveitis, orchitis, and glomerulonephritis.^2^^,^^6^^,^^7^^,^^9^^,^^13^ The presence of many bacilli along with neutrophils, vasculitis, and panniculitis is unique to ENL, and these histopathologic features readily differentiate ENL from other leprosy variants.^2^^,^^3^ However, the density of neutrophils is dependent on chronicity. Older lesions have a greater number of T cells, histiocytes, and plasma cells.^2^

Treatment regimens include systemic corticosteroids, thalidomide, and long-term antibacterial therapy.^1^^,^^5^^,^^9^^,^^14^ In this case, the symptoms of the immunologic reaction temporarily improved due to treatment with systemic steroids; unlike minocycline, doxycycline has no antibacterial activity against *M. leprae*. Thalidomide is Food and Drug Administration-approved for ENL. Its immunomodulatory effect on tumor necrosis factor-α results in greater than 90 percent efficacy; however, teratogenicity, neurotoxicity, cost, and need for follow-up and compliance limit its availability.^2^^,^^6^^,^^9^^,^^15^ Duration of treatment is long for all patients; however, severity at initial presentation correlates with length of treatment.^9^ Steroids are tapered over 3-6 months, while bactericidal agents are continued for 2 years. Methotrexate and thalidomide are often used as steroid-sparing agents. A small percentage of patients with ENL may be resistant to treatment, leading to morbidity or mortality.^6^ ENL is more likely to become recurrent compared to type 1 reactions, sometimes years following treatment.^14^

ENL is a debilitating complication of *M. leprae* or *M. lepromatosis* infection. ENL should be considered for a patient who presents with episodic cutaneous findings in association with fever, lymphadenitis, and neuritis. Although more likely after treatment, immunologic reactions including ENL can manifest before treatment and even before diagnosis of the underlying leprosy subtype (Table I). As the presenting symptoms of LL in a patient without prior clinically apparent skin findings, ENL can create a diagnostic conundrum in areas where leprosy is uncommon. Early diagnosis and treatment can limit transmission of disease and permanent disability.

**Table I:** Cases of ENL presenting without prior history of leprosy diagnosis or treatment

Reference	Cutaneous findings	Extracutaneous features	Histopathology	History of leprosy treatment	Diagnosis
Woldemichael (2021)^3^	Annular plaques and nodules	Diffuse sensorimotor peripheral neuropathy	Numerous bacilli with scattered neutrophils and small vessel vasculitis	No	LL
Quintarelli (2023)^4^	Symmetric erythematous nodules with purulent discharge on the lower extremities; erythematous nodules on the face	Recurrent fevers, arthralgia, oral aphthosis, cervical and axillary lymphadenopathy, and unilateral loss of sensation over the 4th and 5th digits	Granulomatous inflammation with necrosis and suppuration along with acid-fast bacilli	No	LL
Bala (2014)^7^	Nodular, ulcerative, and impetiginous lesions of the trunk, upper extremities, and thighs over 3 months	None	Narrow grenz zone overlying dense dermal infiltrate and numerous acid-fast bacilli, discretely and in clusters	No	LL
Kaushik (2020)^8^	Painful papulonodules on the upper limbs and back, blisters, and ulceration	High-grade fever, bilateral ulnar nerve thickening	Granulomas, nerve destruction, vasculitis, and panniculitis	No	LL
Prabhu (2009)^1^	Recurrent painful, erythematous, nodules for 6 months, which originally presented on the palms and then became widespread	Fever, arthralgias, fatigue, bilateral ulnar nerve thickening, and loss of temperature sensation on the extremities	Edema, endothelial swelling, and perivascular and periadnexal lymphoplasmocytic and neutrophilic infiltrate	No	LL
Maulida (2023)^11^	Multiple ulcers with necrotic bases and granulation on the ears, trunk, and extremities	Madarosis, inguinal lymphadenopathy, thickening of nerves, and numbness in both hands and feet	Necrosis and mixed infiltrate with neutrophils and lymphocytes	No	LL
Rupan (2022)^13^	Annular plaques over the limbs, gluteal region, extensor forearms, arms, and chest; punched-out, tender ulcer present over the glans penis	Fever, bilateral orchitis and inguinal lymphadenopathy, tender pitting pedal edema, ulnar and common peroneal nerve thickening, and bilateral stocking-type anesthesia; ulnar claw hand and foot drop.	Nodular mixed infiltrates including neutrophils associated with edema and vasodilation; perineural and periadnexal infiltration by neutrophils, lymphocytes, and histiocytes	No	BL
Pandhi (2017)^12^	Pustules, nodules, and plaques with ulceration predominantly involving the limbs and trunk	Fever, malaise, multiple thickened peripheral nerves, and recurrent epistaxis	N/A	No	Unspecified leprosy type

*BL*, Borderline leprosy; *ENL*, erythema nodosum leprosum; *LL*, lepromatous leprosy; *N/A*, not applicable.

## Conflicts of interest

None disclosed.
